# Prediction of potential suitable habitat for *Monochasma savatieri* in China under climate change scenarios

**DOI:** 10.3389/fpls.2026.1796816

**Published:** 2026-05-29

**Authors:** Guisheng Luo, Xiaodeng Shi, Suzhen Liu, Yuling Zou, Wei Huang, Huacong Zhang, Lili Shen, Wendi Lan, Baolian Zhang, Wenbiao You, Fengqing Li, Ruixin Liu, Jiaxuan Luo

**Affiliations:** 1 Experimental Center of Subtropical Forestry, Chinese Academy of Forestry, Fenyi, China; 2 Zhejiang Academy of Forestry, Hangzhou, China

**Keywords:** climate change, ecological niche modeling, habitat quality degradation, hemi-parasitic plant, *M. savatieri*

## Abstract

**Introduction:**

*M. savatieri* is a medicinal hemi-parasitic herb with significant therapeutic value and is mainly distributed in southeastern China, yet its development is constrained by limited wild resources under global climate change.

**Methods:**

Using the MaxEnt model, this study simulated the potential distribution of *M. savatieri* in China under current and three future climate scenarios (SSP1‑2.6, SSP2−4.5, SSP5−8.5) and identified the key environmental drivers.

**Results:**

Results show that the current highly suitable habitat (18.56×10^4^ km²) is mainly located in Jiangxi, Zhejiang, Fujian, and surrounding provinces, which lie within the subtropical monsoon climate zone, primarily shaped by four variables: precipitation of the coldest quarter (bio19), precipitation of the driest month (bio14), maximum temperature of the warmest month (bio5), and annual precipitation (bio12), which collectively explain 92.2% of the model. Under all future scenarios, the highly suitable area is projected to decline, contracting notably around central Jiangxi, while the overall distribution centroid remains stable.

**Discussion:**

These findings provide spatially explicit guidance for the conservation of wild resources and the selection of cultivation areas for *M. savatieri* under climate change.

## 1 Introduction

Global warming has emerged as a significant threat to global biodiversity in the 21st century, with nearly a quarter of plant species worldwide at risk of extinction due to climate change (Thomas et al., 2004). The IPCC Sixth Assessment Report projects that the global average temperature will rise by 1.0 °C to 5.7 °C by the century’s end (Masson-Delmotte et al., 2021). This persistent warming will profoundly influence species distribution patterns and physiological-ecological traits (Hu et al., 2015). Numerous studies indicate that global warming will result in habitat fragmentation, loss of distribution ranges, and accelerated biodiversity loss (Cha et al., 2024; Zhang et al., 2024a; Shi et al., 2021). Recent predictions suggest that at 2 °C of global warming, 16% of plant species are projected to lose more than half of their climatically determined geographic ranges (Warren et al., 2018). Consequently, forecasting the potential suitable habitats of species and their migration trends in response to future climate change can provide essential scientific support for the planning of plant introduction and cultivation, as well as for ecological conservation and management strategies.

Species distribution models (SDMs) serve as crucial tools for examining the relationships between species and their environments. They can simulate potential species distributions and their responses to climate change by integrating species distribution data with environmental variables (Yi et al., 2018). Among the various SDMs, the maximum entropy-based MaxEnt model has been extensively utilized in areas such as endangered species conservation (Wang et al., 2020; Alsamadisi et al., 2020; Yang and Huang, 2021), invasive species risk assessment (Gallagher et al., 2010), and ecosystem management and protection (Ma and Pan, 2024). This model is favored for its predictive accuracy, adaptability to small sample sizes, and computational efficiency. As human demands for health increase and reliance on medicinal plants grows, the MaxEnt model has been effectively employed to simulate potential habitat suitability for several medicinal plants, including *Saussurea* species (Zhao et al., 2024), *Angelica dahurica* (Zhang et al., 2024b), and *Epimedium brevicornu* (Liu et al., 2025). Recent studies on Chinese medicinal plants have increasingly addressed methodological challenges in MaxEnt applications, particularly model tuning and variable selection. For instance, optimized MaxEnt models incorporating ENMeval or kuenm packages have significantly improved prediction accuracy for species such as *Changium smyrnioides* (Zhu et al., 2025), *Alpinia officinarum* (Kang et al., 2025), and *Leonurus japonicus* (Wang et al., 2023). These optimized approaches typically involve adjusting the regularization multiplier and selecting appropriate feature combinations to mitigate overfitting and enhance model transferability. Furthermore, systematic variable selection using correlation analysis and contribution assessment has been widely adopted to reduce multicollinearity and improve model parsimony. Key environmental drivers identified in these studies often include temperature-related variables (e.g., bio11, mean temperature of coldest quarter) and precipitation-related variables (e.g., bio14, precipitation of driest month). Collectively, these methodological advancements provide a robust framework for predicting potential habitats of medicinal plants and assessing their vulnerability under future climate scenarios.

*M. savatieri* is a perennial hemi-parasitic herb that belongs to the genus *Monochasma* within the family Orobanchaceae (formerly Scrophulariaceae). This species is primarily found in southern China, particularly in Jiangxi, Hubei, and Fujian (Hong et al., 1998; Yamazaki, 1993). The whole dried plant of *M. savatieri* is extensively utilized in herbal medicine for its properties of clearing heat, detoxifying, cooling blood, and stopping bleeding. Clinically, it is commonly used to treat a variety of conditions, including colds, internal heat and irritability, cough, hematemesis, dysentery, hematochezia, menstrual disorders, rheumatic bone pain, toothache, and mastitis (Shi et al., 2013; Liu et al., 2013). Due to its significant medicinal effects, market demand for this herb has surged. However, as a traditional wild medicinal plant in China, recent surveys have indicated a sharp decline in its wild populations (Zhang et al., 2015). In Japan, *M. savatieri* has even been classified as an endangered species. Some studies attribute the decrease in wild resources of *M. savatieri* to its biological characteristics; specifically, its hemi-parasitic nature and low seed germination rate lead to a diminished natural reproductive capacity (Yang, 2009). Excessive harvesting and habitat destruction have directly contributed to the decline of wild resources, resulting in a significant supply-demand imbalance that severely limits clinical applications. Current research on *M. savatieri* primarily addresses its chemical composition (Zheng et al., 2012), hemi-parasitic traits (Chen et al., 2021), genetic diversity (Yang et al., 2022), tissue culture and rapid propagation (Li et al., 2021), and artificial cultivation (Zhang et al., 2025). However, studies that predict its potential suitable habitats remain limited. Presently, the utilization of *M. savatieri* depends on wild resources, which are insufficient to meet demand. Consequently, artificial introduction and cultivation are urgently required to fulfill market needs. Preliminary cultivation practices suggest that the growth and development of *M. savatieri* are closely associated with climatic factors. Therefore, predicting the potential suitable habitats of *M. savatieri* based on current and future climate conditions in China is of significant importance for resource development and related industrial growth.

Utilizing the distribution data of *M. savatieri*, this study employs ArcGIS tools and the MaxEnt model to forecast its potential distribution patterns in China under both current and future climate scenarios. The research objectives concentrate on the following aspects:

1. What is the distribution pattern of *M. savatieri* in China under current and future climate scenarios?
2. What are the key environmental factors influencing *M. savatieri*?
3. How will climate change affect the future distribution of suitable habitats for *M. savatieri*?

Addressing these questions can provide theoretical support for the strategic planning of artificial introduction and cultivation of *M. savatieri* in China.

## 2 Materials and methods

### 2.1 Distribution data of *M. savatieri*

**Figure 1:**
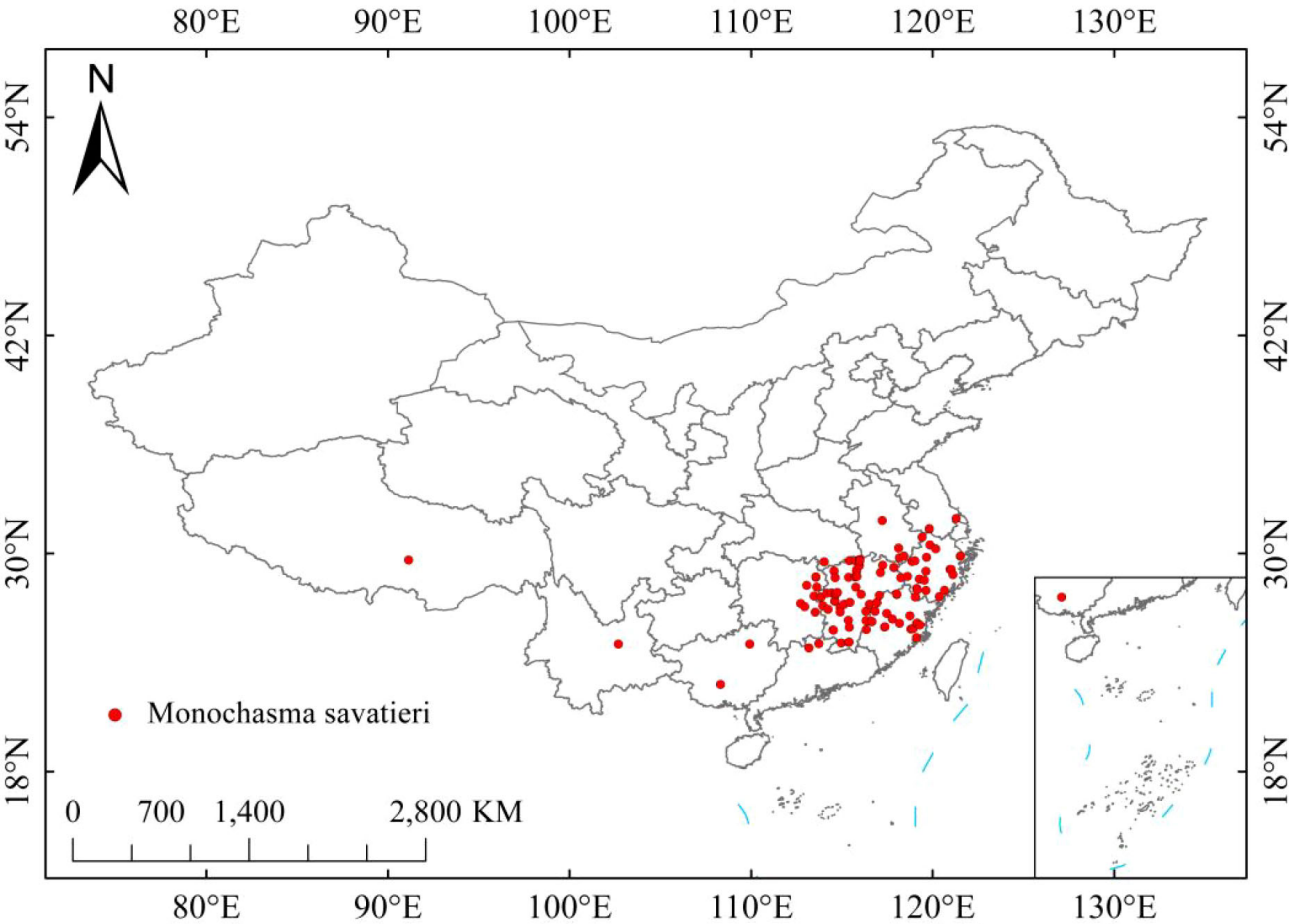
Distribution map of valid occurrence points of *M. savatieri*.

The distribution points of *M. savatieri* were gathered from various sources, including the Chinese Virtual Herbarium (CVH, http://www.cvh.ac.cn), the Global Biodiversity Information Facility (GBIF, https://www.gbif.org), and field surveys conducted by our research team between 2017 and 2024. Specimens that were misidentified, duplicated, or lacked precise geographic information were excluded, resulting in 123 valid distribution records of *M. savatieri* in China. To address potential model overfitting due to spatially clustered points, the distribution data underwent further filtering. Utilizing ArcGIS 10.2, a 5 km × 5 km raster grid was established, ensuring that only one occurrence point was retained per grid cell and that all points were spaced at least 5 km apart. Following this spatial thinning process, 106 valid distribution points remained for modeling. The geographic coordinates of these points were saved in “.csv” format for subsequent analyses (**Figure 1**).

### 2.2 Environmental data acquisition

Climate data variables for the current period (1970–2000) and future periods (2041–2060, 2061–2080, 2081–2100) were retrieved from the WorldClim database (http://www.worldclim.org/). Within the framework of the Sixth Coupled Model Intercomparison Project (CMIP6), three Shared Socioeconomic Pathways (SSPs) were selected: low forcing (SSP1−2.6), medium forcing (SSP2−4.5), and high forcing (SSP5−8.5) (Hurtt et al., 2020). The BCC−CSM2−MR model, developed by the China Meteorological Administration, was utilized. A total of 19 bioclimatic variables and 3 topographic variables—elevation, slope, and aspect—were collected, all at a spatial resolution of 2.5 arc−minutes.

### 2.3 Environmental variable selection

To avoid redundancy in climate data that could lead to model overfitting, we performed correlation screening using Pearson’s correlation coefficient. Multicollinearity analysis was conducted using SPSS 26.0. When the absolute value of the correlation coefficient between two environmental variables exceeded 0.8, the variable with greater biological significance was retained. Finally, 14 variables were selected for modeling with MaxEnt 3.4.4, including bio2, bio3, bio4, bio5, bio6, bio8, bio12, bio14, bio15, bio16, bio18, bio19, slope, and aspect **Table 1**.

**Table 1:** The 22 environmental variables initially acquired.

Code	Variable	Unit
bio1	Annual Mean Temperature	°C
**bio2**	**Mean Diurnal Range**	**°C**
**bio3**	**Isothermality**	**°C**
**bio4**	**Temperature Seasonality**	**—**
**bio5**	**Max Temperature of Warmest Month**	**°C**
**bio6**	**Min Temperature of Coldest Month**	**°C**
bio7	Temperature Annual Range	°C
**bio8**	**Mean Temperature of Wettest Quarter**	**°C**
bio9	MeanTemperature of Driest Quarter	°C
bio10	Mean Temperature of Warmest Quarter	°C
bio11	Mean Temperature of Coldest Quarter	°C
**bio12**	**Annual Precipitation**	**mm**
bio13	Precipitation of Wettest Month	mm
**bio14**	**Precipitation of Driest Month**	**mm**
**bio15**	**Precipitation Seasonality**	**—**
**bio16**	**Precipitation of Wettest Quarter**	**mm**
bio17	Precipitation of Driest Quarter	mm
**bio18**	**Precipitation of Warmest Quarter**	**mm**
**bio19**	**Precipitation of Coldest Quarter**	**mm**
Elevation	Elevation	m
**Slope**		**°**
**Aspect**		**rad**

Variables retained for modeling are indicated in bold.

### 2.4 MaxEnt model parameter settings

In this study, MaxEnt version 3.4.0 was utilized for modeling. The following parameters were configured within the MaxEnt model: a Jackknife test was conducted to evaluate variable importance; response curves were generated; prediction maps were produced; the output format was set to logistic; a random test percentage of 25% was applied, whereby 75% of the distribution points were designated for model construction (training data), and 25% were reserved for model validation (testing data); the regularization multiplier was established at 1. Cross-validation was implemented to address uncertainty arising from the division of training and validation sets. The model was executed 10 times, each time randomly partitioning the species distribution data into 10 subsets, with one subset serving as the validation set and the remaining nine as the training set. The “Random seed” option was activated, and the maximum number of iterations was set to 500, while all other settings remained at their default values. The final outputs were saved in “.asc” format. The predictive performance of the MaxEnt model was assessed using the omission rate and the area under the receiver operating characteristic curve (AUC). AUC values ranging from 0.5 to 0.6, 0.6 to 0.7, 0.7 to 0.8, 0.8 to 0.9, and 0.9 to 1 correspond to model performance classified as “poor,” “fair,” “moderate,” “good,” and “excellent,” respectively.

### 2.5 Classification of suitable habitat grades for *M. savatieri*

The average ASCII data from the 10 MaxEnt model runs were imported into ArcGIS 10.8 for visualization and reclassification. The predicted probability values ranged from 0 to 1, with values closer to 1 indicating a higher likelihood of species presence. The potential distribution area was divided into four grades: Highly suitable area: (0.63, 1]; Moderately suitable area: (0.33, 0.63]; Low suitable area: (0.10, 0.33]; Unsuitable area: ≤0.10.

### 2.6 Spatial pattern changes and centroid analysis of species’ suitable habitats

In ArcGIS, the suitable habitat areas were binarized by setting regions with a distribution probability < 0.1 as unsuitable habitats (assigned a value of 0) and regions with a distribution probability≥0.1 as suitable habitats (assigned a value of 1), thereby obtaining binary maps of unsuitable/suitable habitats for each period. The matrix was defined as follows: 0–0 represents unsuitable areas, 0–1 represents newly suitable areas, 1–0 represents lost suitable areas, and 1–1 represents retained suitable areas. The area changes, trends, and extents of suitable habitats for *M. savatieri* under different climate scenarios compared to the current period were calculated, including the area and geographic range of expansion, retention, and contraction. Based on the binary maps, the SDM toolbox was used to calculate the changes in the geometric center of potential suitable habitats across different periods, and the overall migration trends of the core suitable habitats of *M. savatieri* were compared, reflecting the impact of environmental changes on its distribution across different periods.

## 3 Results and analysis

### 3.1 Model performance and key environmental factors

**Figure 2:**
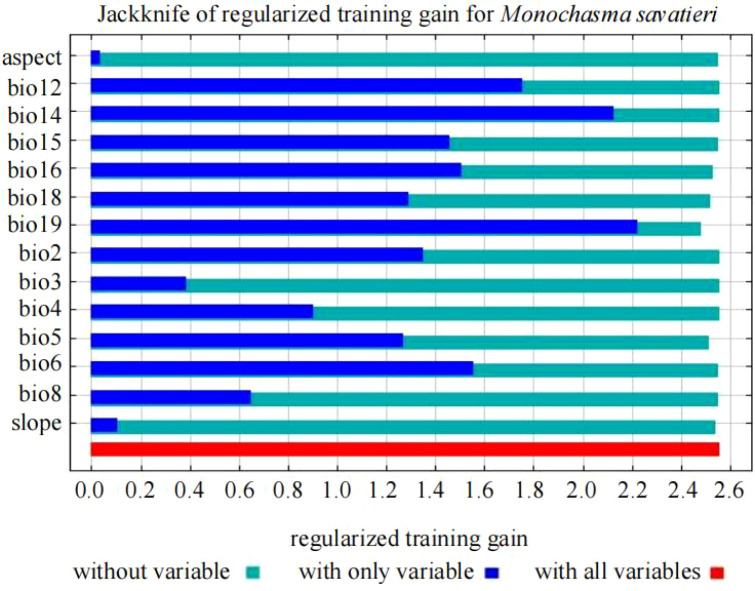
Importance of each variable in training data assessed by jackknife test.

**Figure 3:**
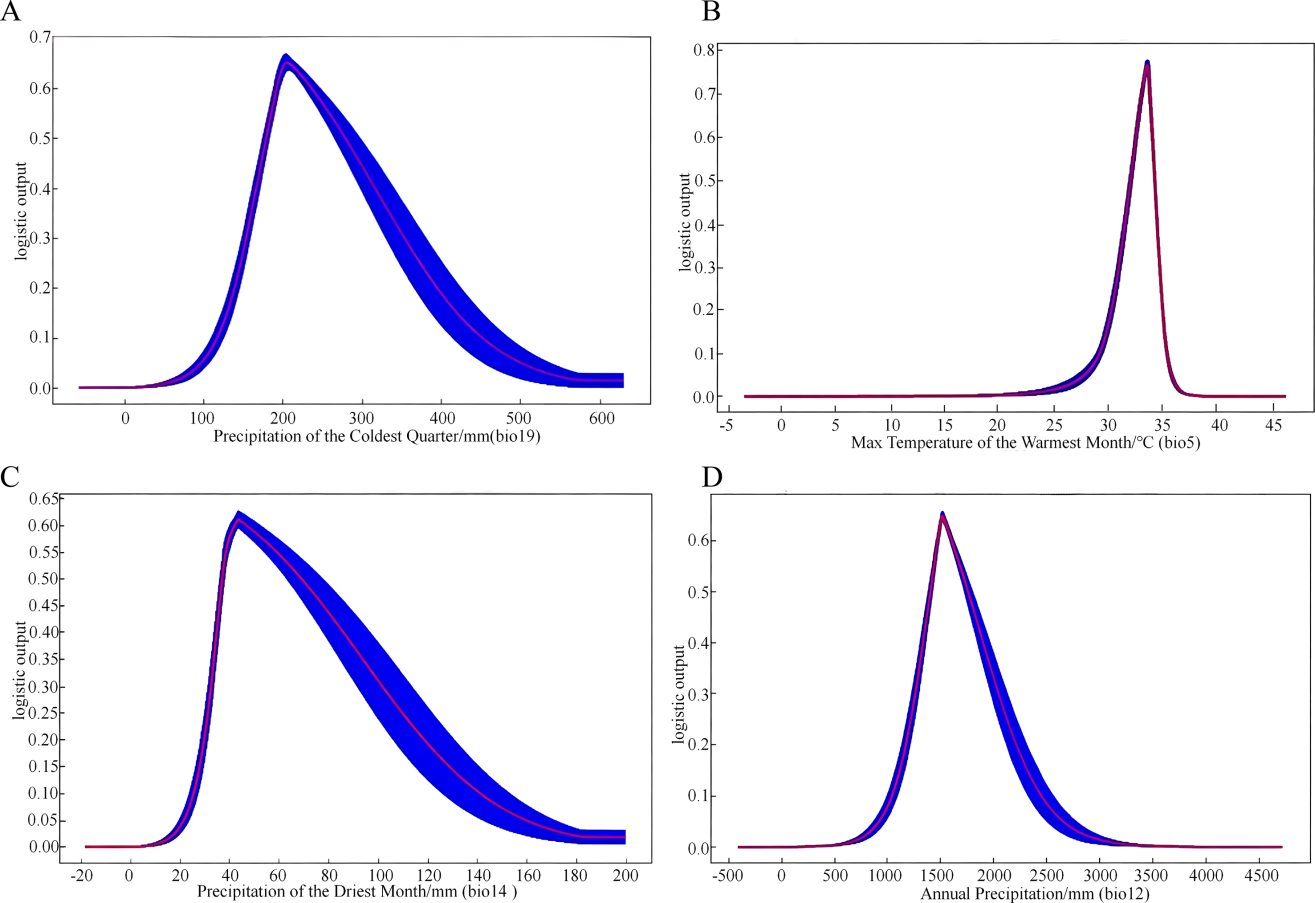
Response curves of dominant environmental variables to the habitat suitability probability of *M. savatieri* (logistic output represents the probability of species suitability, with higher values indicating stronger suitability; the red line is the model fitting curve, and the blue area represents the confidence interval). **(A)** Precipitation of the coldest quarter (bio19); **(B)** Max temperature of the warmest month (bio5); **(C)** Precipitation of the driest month (bio14); **(D)** Annual precipitation (bio12).

Under the current climatic conditions, the ROC curves output by the MaxEnt model show that the training and test AUC values of the current geographic distribution model, constructed based on 14 environmental factors, are 0.976 ± 0.001 and 0.965 ± 0.006, respectively, indicating that the model performs well and has high reliability. Among the 14 environmental variables incorporated into the model, the three most significant contributors were bio19 (Precipitation of the Coldest Quarter), bio5 (Max Temperature of the Warmest Month), and bio14 (Precipitation of the Driest Month), which collectively accounted for approximately 92.2% of the model’s predictive contribution (**Table 2**). The Jackknife test further revealed that bio19 (Precipitation of the Coldest Quarter), bio12 (Annual Precipitation), and bio14 (Precipitation of the Driest Month) were the most critical environmental factors affecting the distribution of *M. savatieri* **Figure 2**. In summary, the primary environmental determinants of *M. savatieri* distribution includes bio19 (Precipitation of the Coldest Quarter), bio5 (Max Temperature of the Warmest Month), bio14 (Precipitation of the Driest Month), and bio12 (Annual Precipitation). Based on the response curves of the dominant environmental variables (**Figure 3**), the optimal habitat conditions for *M. savatieri* are as follows: Precipitation during the coldest quarter (bio19) ranges from 179.2 to 278.6 mm. Precipitation in the driest month (bio14) varies between 37.1 and 68.9 mm. The maximum temperature recorded in the warmest month (bio5) is between 32.1 and 34.2 °C. Annual precipitation (bio12) amounts to between 1406.00 and 1754.67 mm.

**Table 2:** Contribution of environmental factors influencing the distribution of *M. savatieri*.

Variable	Percent contribution	Permutation importance
bio19	82.4	53.6
bio5	4.9	9.9
bio14	4.9	0.1
slope	2.3	0.3
bio4	1.8	0.1
bio6	0.9	2.9
bio18	0.8	26.4
bio2	0.7	1
bio16	0.5	3.9
bio15	0.3	0.8
bio3	0.2	0.3
aspect	0.2	0.1
bio8	0.1	0.6
bio12	0	0

### 3.2 Prediction of the potential geographical distribution of *M. savatieri* under current climatic conditions

**Figure 4:**
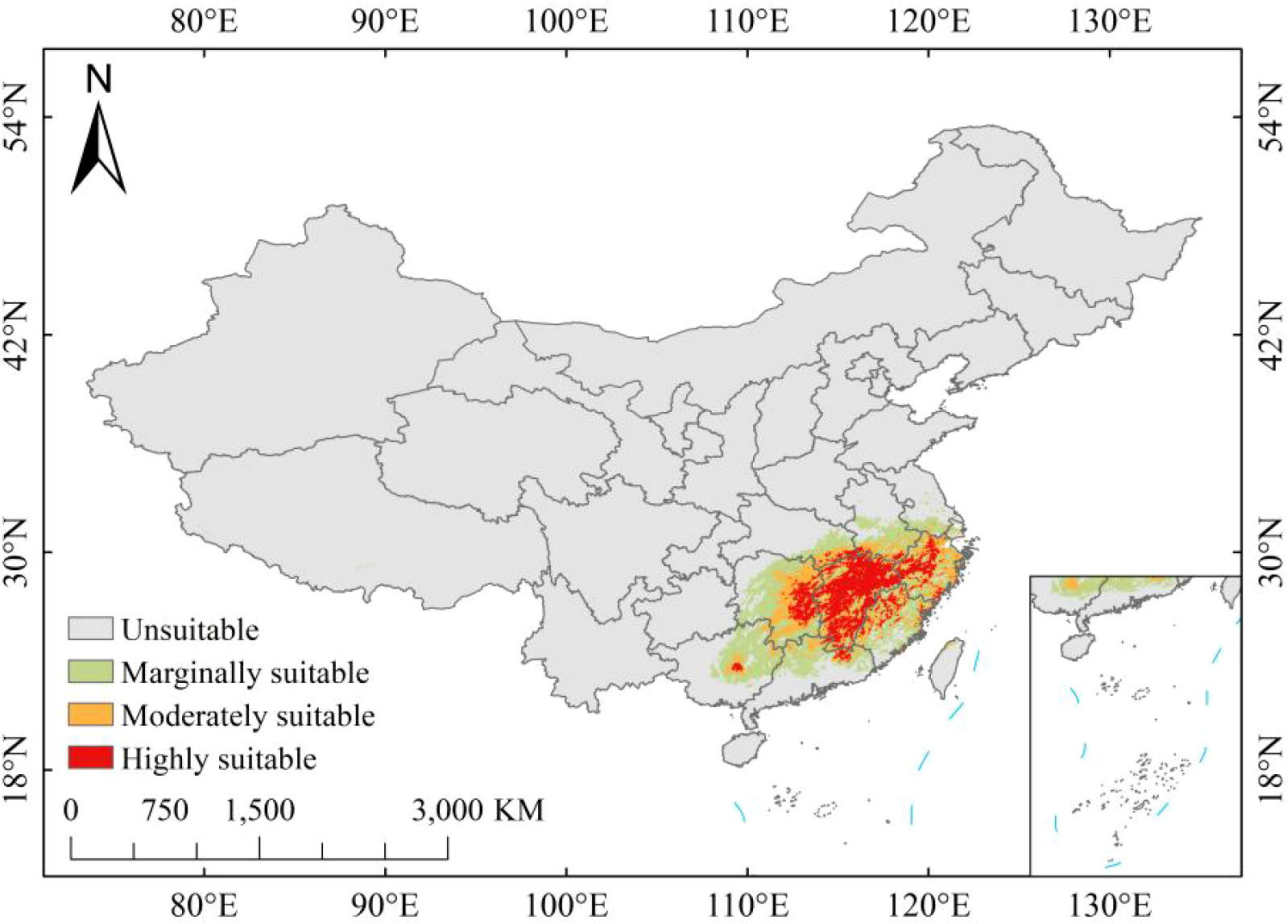
Current potential distribution of *M. savatieri*.

Based on the results of the MaxEnt model, Geographic Information Systems (GIS) were utilized to delineate and visualize potential suitable habitats for *M. savatieri* across different periods, as depicted in **Figure 4**. The red, yellow, green, and gray regions indicate high, moderate, low, and unsuitable habitat areas, respectively. **Figure 4** reveals that the predicted potential distribution of *M. savatieri* in China encompasses nine provinces and municipalities, primarily situated in southeastern China, with a total suitable area of approximately 74.55 × 10^4^ km². The species is predominantly found in Jiangxi, Zhejiang, Fujian, Hunan, southern Hubei, southern Anhui, southern Jiangsu, northern Guangdong, and northeastern Guangxi. Among these regions, the highly suitable area spans approximately 18.56 × 10^4^ km², which constitutes about 7.77% of the national land area, with core areas concentrated in Jiangxi, Zhejiang, Fujian, southeastern Hunan, southeastern Hubei, southern Anhui, northern Guangdong, and central Guangxi. Conversely, the remaining provinces and regions are classified as unsuitable habitats for *M. savatieri*, encompassing a total area of approximately 885.45 × 10^4^ km², representing about 92.23% of the national territory.

### 3.3 Potential suitable habitat of *M. savatieri* under future climate conditions

**Figure 5:**
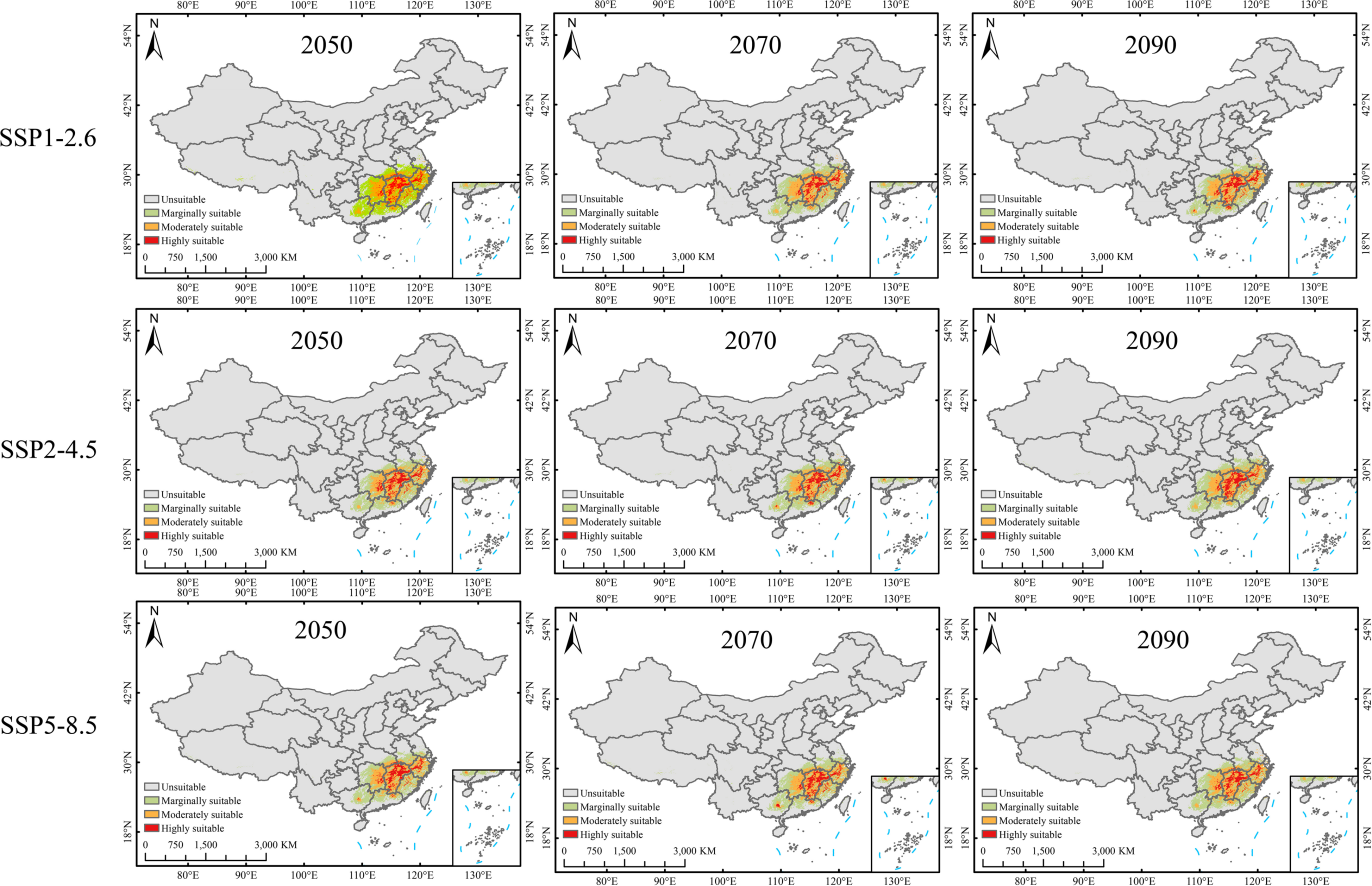
Potential distribution of *M. savatieri* based on different future climate scenarios.

**Figure 6:**
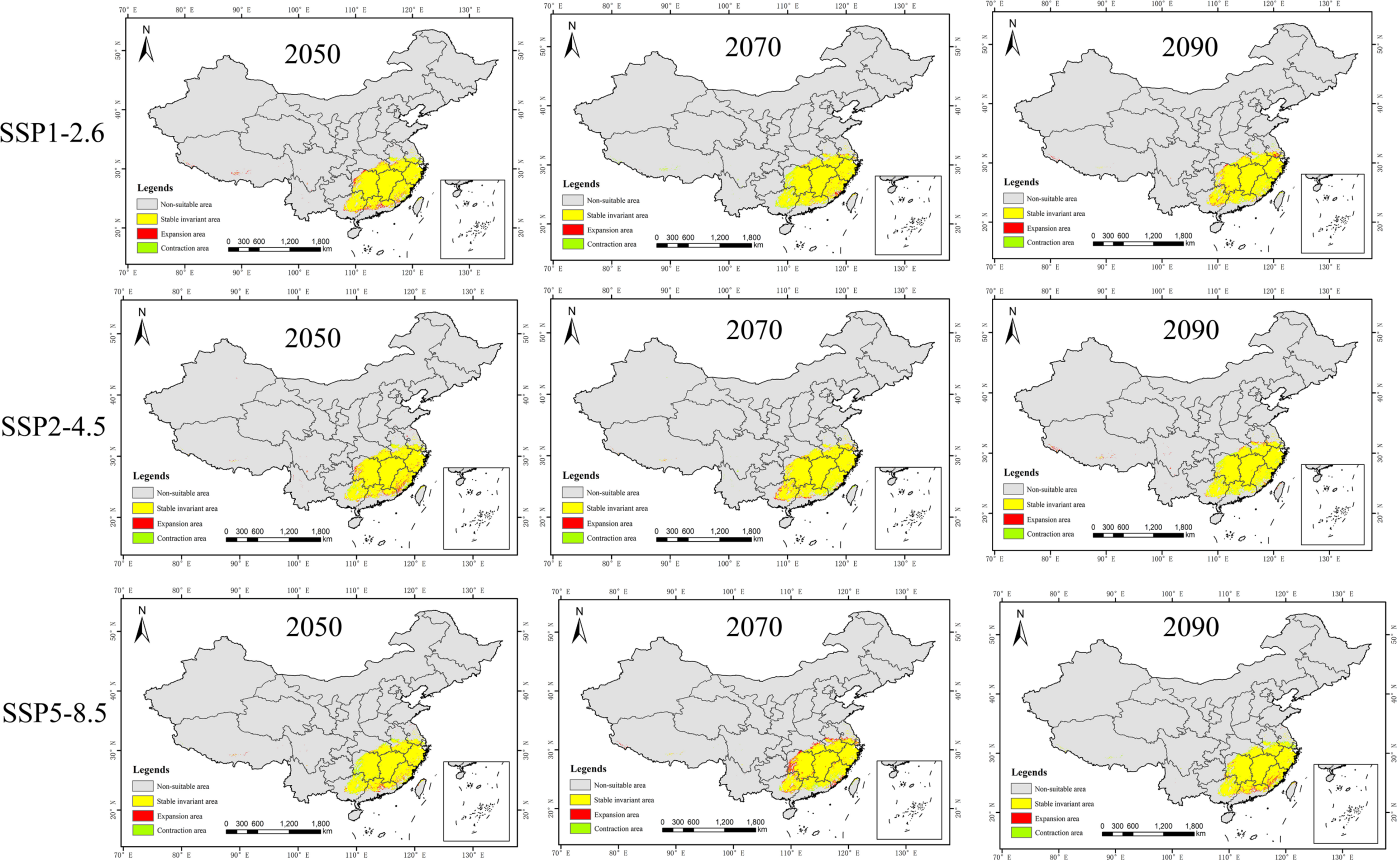
Trends in changes of suitable habitat areas under different climate scenarios.

Following the same criteria described above, the MaxEnt model was used to predict the potential suitable habitat of *M. savatieri* for the 2050s, 2070s, and 2090s under the SSP1-2.6, SSP2-4.5, and SSP5-8.5 scenarios. We obtained spatial distribution maps of the predicted potential suitable areas (**Figure 5**), maps showing dynamic changes in suitability grades (**Figure 6**), and the area and changes of each suitability class (**Table 3**).

**Table 3:** Prediction area of potential suitable distribution area in different periods 10^4^km^2^.

Period	current	2050s			2070s			2090s		
		SSP126	SSP245	SSP585	SSP126	SSP245	SSP585	SSP126	SSP245	SSP585
Marginally suitable	35.66	40.26	38.91	36.96	38.79	40.50	40.94	39.25	39.79	39.32
Moderately suitable	20.33	28.30	28.35	27.05	26.10	27.51	26.70	25.92	25.63	27.40
Highly suitable	18.56	7.84	7.76	8.32	8.61	7.52	8.25	8.44	8.48	7.85
Total suitable area	74.55	76.40	75.02	72.33	73.50	75.53	75.89	73.61	73.90	74.57
Compare with the current increase		1.85	0.47	-2.22	-1.05	0.98	1.34	-0.94	-0.65	0.02

Under future climate scenarios, the total suitable area for *M. savatieri* exhibits varying degrees of change relative to the present period. By the 2050s, under the SSP1-2.6 and SSP2-4.5 scenarios, the total suitable area increases by 1.85×10^4^ km² and 0.47×10^4^ km², respectively, with newly suitable regions predominantly located in northern Hunan, central Guangdong, and northern Fujian. In contrast, under the SSP5-8.5 scenarios, the total area declines by 2.22×10^4^ km², primarily due to habitat loss in western Hunan and parts of eastern Guangxi.

The area of highly suitable habitats exhibits a declining trend across all three climate scenarios, while areas classified as moderately and low suitable habitats are increasing. The most pronounced reduction in highly suitable area occurs under the SSP2-4.5 scenarios, with a decrease of 7.76×10^4^ km², compared with the current high-suitability area of 18.56 ×10^4^ km², this represents a decrease of 57.70%. This change is primarily due to the conversion of highly suitable areas in Jiangxi, Zhejiang, Fujian, southeastern Hunan, southeastern Hubei, southern Anhui, northwestern Guangdong, and portions of central Guangxi into moderately suitable areas.

By the 2070s, under the SSP2-4.5 and SSP5-8.5 scenarios, the total suitable area is projected to increase by 0.98×10^4^ km² and 1.34×10^4^ km², respectively. These expansions are expected to occur in central Guangxi, central Guangdong, as well as in northwestern Hunan, southern Hubei, and southern Henan. In contrast, the SSP1-2.6 scenarios predict a decrease in the total area by 1.05×10^4^ km², primarily affecting central Guangxi, northwestern Hunan, and southern Jiangsu. The trends in changes across suitability grades remain consistent with those observed in the 2050s, with the SSP2-4.5 scenarios continuing to exhibit the most significant reduction in highly suitable area—a decline of 7.52×10^4^ km² (Compared with the current high-suitability area of 18.56 × 10^4^ km², this represents a decrease of 57.70%.). The spatial pattern of transition mirrors that described for the 2050s.

By the 2090s, under the SSP126 and SSP245 scenarios, the total suitable area decreases by 0.94×10^4^ km² and 0.65×10^4^ km², respectively. These reductions primarily occur in central Guangdong, parts of Fujian, northern Hunan, central Guangxi, and additional areas within central Guangdong. In contrast, the SSP5-8.5 scenarios indicate no significant change in the total area. The trends in changes to suitability grades continue to reflect the patterns observed in earlier periods. Notably, the SSP5-8.5 scenarios demonstrate the most substantial reduction in highly suitable areas, declining by 7.85×10^4^ km², compared with the current high-suitability area of 18.56 × 10^4^ km², this represents a decrease of 57.70%. The principal transition regions remain consistent with those identified in previous periods, predominantly located in Jiangxi, Zhejiang, Fujian, southeastern Hunan, southeastern Hubei, southern Anhui, northwestern Guangdong, and central Guangxi.

### 3.4 Shifts in the centroid of highly suitable habitats for *M. savatieri* under different climate conditions

**Figure 7:**
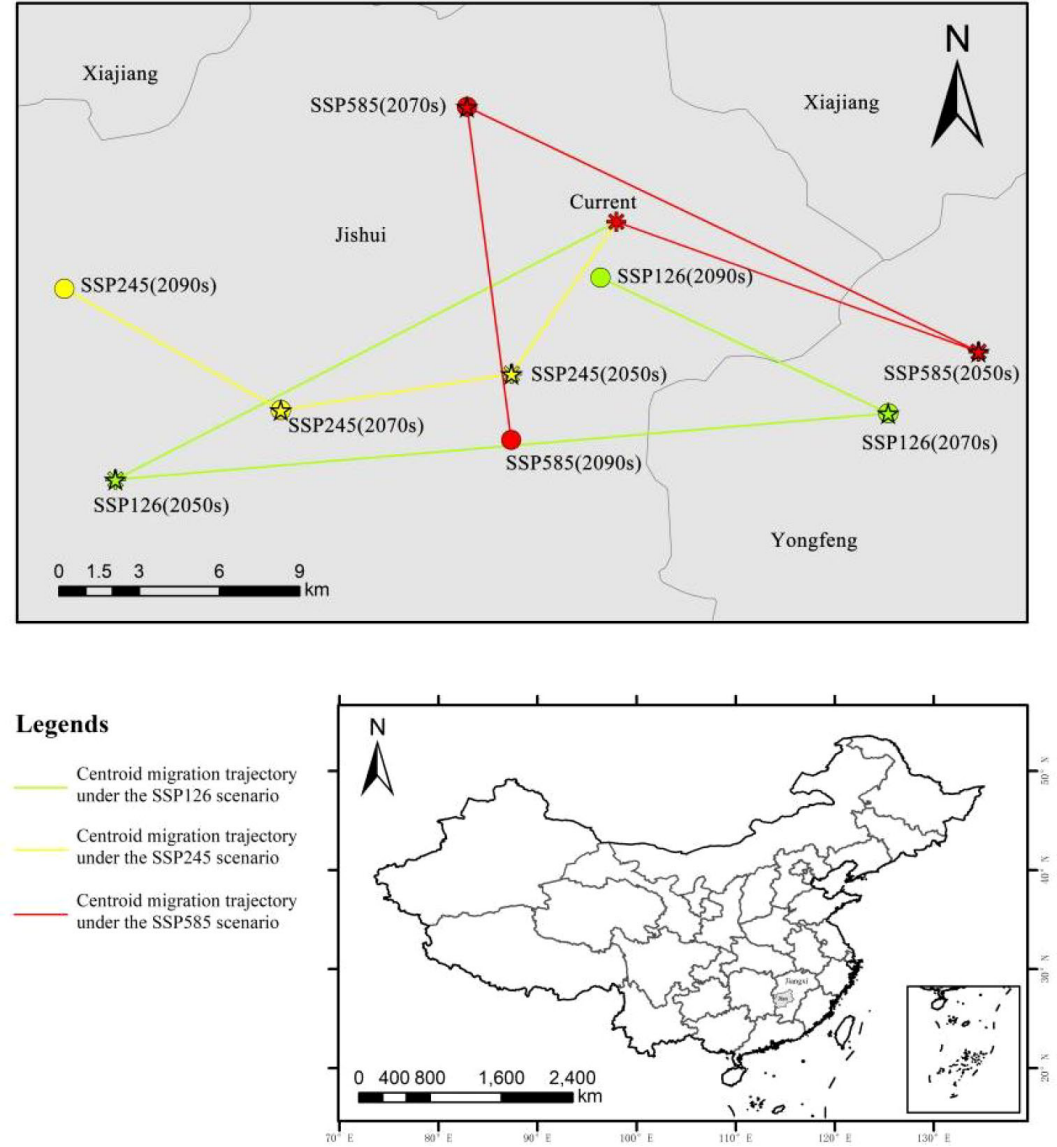
Migration trajectories of the centroid of potential distribution for *M. savatieri* under different climate scenarios.

Highly suitable habitats for each period were delineated using ArcGIS software, and the centroid positions of these habitats were computed. As illustrated in **Figure 7**, under current climatic conditions, the centroid of the highly suitable area for *M. savatieri* is situated in Jishui County, Jian City, Jiangxi Province (27.428°N, 115.264°E). Under the SSP1-2.6 scenarios, the centroid shifts to 27.341°N, 115.096°E in the 2050s; moves to 27.364°N, 115.356°E in the 2070s; and is located at 27.409°N, 115.259°E in the 2090s. Compared to the present period, the centroid exhibits a general pattern of first moving southwest, then east, and finally northwest, with an overall displacement distance that remains relatively small. Under the SSP2-4.5 scenarios, the centroid is positioned at 27.377°N, 115.229°E in the 2050s; at 27.365°N, 115.151°E in the 2070s; and at 27.406°N, 115.078°E in the 2090s.

The overall movement direction is westward compared to the present. Under the SSP585 scenarios, the centroid is located at 27.384°N, 115.386°E in the 2050s; at 27.467°N, 115.214°E in the 2070s; and at 27.355°N, 115.229°E in the 2090s. Relative to the current period, the trajectory initially shifts southeast, subsequently northwest, and ultimately south. Overall, across all three climate scenarios, the centroids of highly suitable habitats remain within the boundary region between Jishui County and Yongfeng County, exhibiting relatively small displacement distances.

## 4 Discussion

### 4.1 Major environmental variables affecting the distribution of *M. savatieri*

Temperature and precipitation are critical bioclimatic determinants of plant growth, with different species exhibiting distinct requirements for hydrothermal conditions. Drought and elevated temperatures typically diminish plant water-use efficiency (Punyasena et al., 2008; Bradie and Leung, 2017). The MaxEnt-based analysis conducted in this study indicates that the potential geographical distribution of *M. savatieri* is primarily influenced by the precipitation of the coldest quarter (bio19), the maximum temperature of the warmest month (bio5), the precipitation of the driest month (bio14), and annual precipitation (bio12). The climatic thresholds for suitable habitats are as follows: precipitation of the coldest quarter ranges from 179.2 to 278.6 mm, precipitation of the driest month ranges from 37.1 to 68.9 mm, maximum temperature of the warmest month ranges from 32.1 to 34.2 °C, and annual precipitation ranges from 1406.00 to 1754.67 mm. These findings are consistent with studies on other medicinal plants, such as *Epimedium brevicornu* (Liu et al., 2025) and *Coptis chinensis* (Li et al., 2020), which also emphasize water availability and temperature as the primary factors influencing their distribution.

The results further indicate that the distribution of *M. savatieri* is influenced not only by total annual precipitation but also by water availability during dry and cold periods, while demonstrating a distinct upper thermal limit in summer. This ecological adaptation is intricately linked to its root water-uptake strategy. In natural settings, the species is commonly found on sun-exposed slopes or beneath *Pinus massoniana* forests, characterized by shallow roots and a hemi-parasitic habit. When deprived of host plants and subjected to extreme heat and drought, its shoots are susceptible to desiccation and mortality (Zhang et al., 2015). Consequently, even if annual precipitation remains constant, the highly suitable areas for this species may diminish due to water stress if future drought events become more frequent or if seasonal precipitation patterns change.

It should be noted that **Table 2** shows the percent contribution of bio19 (Precipitation of the Coldest Quarter) is much higher (82.4%) than that of the other variables, while the permutation importance of bio18 (Precipitation of the Warmest Quarter) is also notably high (26.4%). This inconsistency suggests that, despite using a correlation threshold of |r| < 0.8 for variable screening, a strong residual collinearity still exists between these two precipitation variables. The ecological interpretation is that the distribution of *M. savatieri* is primarily limited by winter precipitation (bio19), whereas summer precipitation (bio18) provides partially overlapping but non-redundant information in the model. From a methodological perspective, this implies that percent contribution may be “inflated” for a dominant variable due to the correlational structure among variables, whereas permutation importance better reflects the independent contribution of each variable. Previous studies have demonstrated that multicollinearity among environmental covariates poses significant challenges to reliable variable selection in species distribution modeling (Farrell et al., 2019). The regularization mechanism in MaxEnt can partially mitigate the effects of collinearity (Farrell et al., 2019), but residual correlations may still lead to discrepancies between different importance metrics. Percent contribution and permutation importance are based on fundamentally different calculation principles, which explains why they often yield divergent rankings for correlated variables (biomod2 development team, 2012). Future studies should consider adopting more stringent variable selection methods, such as Variance Inflation Factor (VIF) analysis (Schnase and Carroll, 2022), or using regularization paths to further decouple correlated variables (Shi et al., 2025a). Optimized modeling frameworks incorporating systematic variable screening and parameter tuning have been shown to enhance model reliability and transferability (Shi et al., 2025b). This study offers significant ecophysiological insights into the distribution dynamics of *M. savatieri* in the context of climate change.

### 4.2 Changes in the suitability distribution of *M. savatieri*

This study integrates the MaxEnt model with GIS spatial analysis to demonstrate that the potential suitable habitats for *M. savatieri* in China are both highly concentrated and limited in extent. The total suitable area comprises only approximately 7.77% of the national land area, primarily located in southeastern provinces such as Jiangxi, Zhejiang, and Fujian. This spatial distribution closely corresponds to the species’ stringent ecological requirements for specific hydrothermal conditions, including precipitation during the coldest quarter and driest month, as well as high temperatures in the warmest month. Consequently, the distribution of *M. savatieri* is predominantly influenced by the seasonal hydrothermal regime characteristic of the East Asian monsoon region. Notably, the area deemed highly suitable, which constitutes merely about 1.94% of the national area, displays a more fragmented distribution, underscoring the narrow ecological niche of this species. This localized and fragmented distribution pattern indicates that *M. savatieri* may be particularly susceptible to climate change, including altered precipitation patterns and extreme heat events, as well as to human-induced habitat loss and disturbance. Therefore, the identified highly suitable areas, particularly core contiguous regions such as the junction of Jiangxi, Zhejiang, and Fujian, should be prioritized for *in-situ* conservation and population monitoring. Future research and management strategies must concentrate on the dynamics and sustainability of these critical habitats.

Climate change is anticipated to modify the geographical distributions of plant species, with most alterations linked to rising temperatures during the growing season and decreased precipitation in the future (Stocker et al., 2014). Temperature is the primary driver of a species’ north-south (latitudinal) distribution, whereas precipitation largely determines its east-west (longitudinal) distribution (Zhang et al., 2020). This study simulated changes in the suitable habitat of *M. savatieri* under three future climate scenarios (SSP1-2.6, SSP2-4.5, SSP5-8.5) for the 2050s, 2070s, and 2090s. The results indicate that while the total suitable area fluctuates modestly across scenarios and time periods—showing slight increases or decreases in some instances—the highly suitable area demonstrates a significant and consistent decline across all scenarios and time frames, with a maximum reduction of approximately 59% compared to the current area. This change is primarily driven by the transformation of highly suitable areas in the current core distribution regions—specifically Jiangxi, Zhejiang, Fujian, and adjacent provinces—into moderately suitable areas, suggesting that future climate change may not result in a dramatic contraction of the species’ distribution range but could lead to widespread degradation in habitat quality. The most critical finding of this study is that under future climate conditions, the total suitable area remains stable while the highly suitable area declines sharply, revealing a more insidious threat—severe degradation of habitat quality. Compared with a drastic contraction of distribution range, habitat quality decline typically occurs earlier and is more difficult to detect, as it may lead to population fragmentation, reduced gene flow, and decreased environmental carrying capacity even before range contraction becomes evident. Highly suitable areas represent regions of population growth, high genetic diversity, and strong self-sustainability; when these areas degrade into moderately or low suitable areas, populations become fragmented and long-term viability is compromised. This finding has important implications for conservation and cultivation strategies: rather than focusing solely on overall range changes, priority should be given to identifying and protecting highly suitable areas as “climate refugia,” along with proactive monitoring and restoration of habitat quality. In cultivation practice, resources should not be spread thinly across large total suitable areas but instead concentrated on the fine-scale management of highly suitable areas and ecological restoration of degraded zones, while selecting drought-tolerant, phenologically matched host plants for companion cultivation to mitigate the impacts of habitat quality decline on the long-term persistence of the species.

The reduction in highly suitable habitat predicted by the model may be attributed, on the one hand, to anticipated decreases in precipitation (IPCC, 2019). Reduced precipitation not only lowers the amount of water available to plants but also exacerbates physiological drought stress by decreasing soil moisture. Meanwhile, rising summer temperatures and increased transpiration, combined with inadequate water supply, may induce xylem embolism in plants (Zhao et al., 2013), directly affecting the growth and development of *M. savatieri* and even leading to mortality. On the other hand, unlike typical autotrophic plants, *M. savatieri* possesses a hemiparasitic nature, characterized by an underdeveloped and relatively shallow root system that requires obtaining water and partial nutrients from host plants. Common hosts include *Loropetalum chinense*, *Digitaria sanguinalis*, and *Gardenia jasminoides.* This trait makes the species more sensitive to future climate change. A reduction in growing season precipitation (e.g., bio18) may not only intensify water stress on host plants (such as *Loropetalum chinense* and *Digitaria sanguinalis*), but may also restrict host plant growth, cause range contraction, or alter host phenology, thereby indirectly cutting off the nutrient and water supply to *M savatieri*. Furthermore, the future trend toward aridification (Sillmann et al., 2013; Dyderski et al., 2018) is expected to intensify with increasing radiative forcing concentrations. Winter warming may disrupt the seed dormancy rhythm of *M. savatieri*, leading to seedling exposure to “late spring frost” damage or phenological mismatches with host plants—for example, host plants may senesce prematurely due to drought stress, breaking the parasitic connection, or changes in host root exudates under drought stress at the time of seed germination may limit germination success. These physiological and ecological mechanisms collectively explain why the area of highly suitable habitat declines sharply rather than merely shifting geographically. Therefore, in future cultivation and introduction efforts, water management should be a primary focus, and priority should be given to selecting drought-tolerant, phenologically matched host plants for companion cultivation to enhance the survival rate and overall fitness of *M. savatieri*.

From the perspective of spatial evolution, changes in suitable habitats exhibit distinct characteristics of geographic replacement and local shifts. While highly suitable areas in the current core region gradually diminish, moderately and poorly suitable areas expand in northern Hunan, western Guangdong, and Guangxi. This pattern of “quality decline with minor range migration” suggests that *M. savatieri* may possess a certain adaptive potential to climate change; however, its optimal habitat conditions are contracting as the climate evolves. This finding is consistent with the results for Pulsatilla chinensis (Wu et al., 2025). Nevertheless, an exclusive focus on changes in total area may obscure the ecological risks associated with significant habitat quality deterioration. Utilizing centroid shift modeling, this study further reveals that the response of highly suitable habitats of *M. savatieri* to future climate change demonstrates strong spatial “stationarity” and “limited mobility”.

Centroid coordinates exhibit fluctuations across the three climate scenarios; however, their movement is largely restricted to a small region near the border of Jishui County and Yongfeng County in Ji’an City, Jiangxi Province, with latitude and longitude variations not exceeding 0.1°. The overall displacement distance remains limited. This finding supports the previous conclusion that, while the area deemed highly suitable experiences a significant decline, the total suitable range remains relatively stable. Notably, a single geometric centroid is inherently limited for summarizing large−area suitable habitats, and the observed trajectory may partly be influenced by shifts in peripheral boundaries. Nonetheless, in this study we use centroid shifts only as a descriptive metric of the overall directional trend of the suitable area’s geometric center. Collectively, these results suggest that future climate change may not precipitate large-scale geographic shifts in suitable habitats for this species. Instead, it may lead to a reorganization of habitat quality and a local contraction within the existing core distribution areas.

### 4.3 Limitations of this study

Several limitations in this study, when predicting the distribution pattern of *M. savatieri* in China, collectively introduce important discrepancies between the “potential suitable areas” presented and the actual suitable habitats. First, the MaxEnt model is inherently data−dependent, and this study relied solely on distribution records from within China, which may not adequately represent the species’ full physiological tolerance range (Feeley and Silman, 2011), thereby underestimating its capacity to adapt to broader climatic conditions. Second, regarding climate data, only a single global climate model (BCC−CSM2−MR) was used, failing to account for the substantial uncertainty among different models, which may overestimate or underestimate the magnitude of future range changes; moreover, the regularization multiplier was left at the default value of 1 without species−specific tuning, potentially leading to model overfitting or underfitting. Most critically, *M. savatieri* is a hemiparasitic plant whose distribution is strongly correlated with the presence of its host plants; however, this study focused exclusively on the impacts of climate and topography, neglecting interspecific interactions (Wisz et al., 2013), human activities (Mou et al., 2025), and other biotic factors. This means that areas predicted as highly suitable may in fact be located in regions where the species cannot actually survive, either due to a lack of host plants or because they are heavily urbanized, agricultural, or deforested. Furthermore, as a medicinal plant, the highly suitable distribution areas of *M. savatieri* may not coincide with the regions where its active compound content (and thus medicinal efficacy) is highest. Therefore, the outputs of this study should be interpreted as the upper limit of potential distribution under climatic and topographic constraints, rather than as actual, colonizable suitable habitats. For practical conservation and cultivation planning, it is essential to overlay additional layers such as land use type, human disturbance intensity, existing vegetation cover, and host plant distribution for secondary filtering. Future research should integrate multi−model climate ensembles (e.g., CMIP6 multi−model means), conduct sensitivity tests on the regularization multiplier, incorporate human activity factors (e.g., land use/cover change, Song and Liu, 2019) and host plant availability, and, most importantly, combine the analysis with the variation in active compound concentrations across the distribution range to bridge the gap between potential and actual distributions, thereby providing more reliable guidance for the cultivation zoning and conservation strategies of *M. savatieri*.

Despite these limitations, the MaxEnt model has effectively predicted the potentially suitable distribution areas of *M. savatieri* in China. Future research will seek to address these constraints to enhance the distribution model, thereby offering more accurate guidance for the conservation and cultivation of *M. savatieri.*

## 5 Conclusion

This study employs MaxEnt model analysis to demonstrate that the precipitation during the coldest quarter (bio19), precipitation of the driest month (bio14), maximum temperature of the warmest month (bio5), and annual precipitation (bio12) are the primary environmental factors influencing the distribution of *M. savatieri*. Under current climatic conditions, the potential suitable habitat for *M. savatieri* in China encompasses nine provinces and municipalities, totaling approximately 74.55 × 10^4^ km². This suitable habitat is predominantly located in Jiangxi, Zhejiang, Fujian, Hunan, southern Hubei, southern Anhui, southern Jiangsu, northern Guangdong, and northeastern Guangxi. Among these regions, the highly suitable area accounts for about 18.56 × 10^4^ km², representing approximately 7.77% of the national land area. Under future climate scenarios, the suitable habitat for *M. savatieri* is expected to undergo systematic structural changes: the area of highly suitable habitats will experience a widespread and consistent decline, while areas classified as moderately and low suitable will increase correspondingly. This shift is primarily driven by the transformation of highly suitable zones in the original core distribution regions, such as Jiangxi, Zhejiang, and Fujian, into moderately suitable areas. Although the total suitable range shows limited fluctuation across different scenarios, this transition leads to an overall degradation of habitat quality. The reduction in highly suitable area is most pronounced under the medium-emission scenarios (SSP2-4.5). This research offers theoretical support for the *in-situ* conservation and artificial cultivation of *M. savatieri.*

## Data Availability

Publicly available datasets were analyzed in this study. This data can be found here: Chinese Virtual Herbarium (CVH, http://www.cvh.ac.cn), the Global Biodiversity Information Facility (GBIF, https://www.gbif.org).
